# The influence of sample biases on estimations of marine microbial diversity

**DOI:** 10.1186/1471-2105-13-S12-A5

**Published:** 2012-07-31

**Authors:** Caroline Rempe, Charles R Budinoff, T Chad Effler, Alison Buchan

**Affiliations:** 1 Genome Science and Technology, The University of Tennessee, Knoxville, TN 37916, USA; 2 Microbiology, The University of Tennessee, Knoxville, TN 37916, USA

## Background

The *Roseobacter* clade is a widespread, metabolically versatile, and easily cultured lineage of marine microbes. Furthermore, cultured isolates are generally considered to be representative of the naturally occurring diversity in the environment. *Roseobacter* is thus considered an ideal clade for exploring and understanding microbial function in a representative model system. Nevertheless, recent studies question whether cultured strains are truly representative of natural populations. Support for one of these studies was based on the potentially biased samples of a Global Ocean Sampling (GOS) expedition [1]. The marine metagenomic studies of the GOS expeditions initially focused on microbes collected from a single filter range (0.1um – 0.8um) and it has been previously suggested that this excludes many *Roseobacters*, thus biasing the dataset towards smaller cells.

## Materials and methods

Here, we take advantage of a GOS metagenomic dataset from a 2006-2008 Antarctic expedition that included community analyses over three different size classes (0.1um-0.8um; 0.8um-3um; and 3um-200um). We examined correlations between bacterial taxonomic diversity (based on 16S rRNA gene analyses) and the three size classes to understand the potential for diversity biases between filter ranges. The 16S rRNA gene sequences from these datasets were extracted using Metaxa and subsequently analyzed using MOTHUR, which provided fine-level resolution taxonomic assignments.

## Results

This analysis reveals a potential bias as the ranges of *Roseobacter* subgroup diversity in this dataset varied between size classes. From this analysis we identified specific *Roseobacter* subgroups that were predominantly found in specific size classes. For instance, the OCT and *Roseovarius* subgroups were most prevalent in the 0.8um-3um filter range, while the DC5-80 and NAC11-7 subgroups were well represented in both 0.1um-0.8um and 0.8um-3um filter ranges. These findings reveal the value of analyzing multiple size class fractions when assessing microbial diversity.
